# A randomised placebo-controlled, double-blind phase II study to explore the safety, efficacy, and pharmacokinetics of sonlicromanol in children with genetically confirmed mitochondrial disease and motor symptoms (“KHENERGYC”)

**DOI:** 10.1186/s12883-022-02685-3

**Published:** 2022-04-27

**Authors:** Jan Smeitink, Rob van Maanen, Lonneke de Boer, Gerrit Ruiterkamp, Herma Renkema

**Affiliations:** 1 Khondrion BV, Transistorweg 5C, M Building, 6534 AT Nijmegen, The Netherlands; 2 Radboud Center for Mitochondrial Medicine, Department of Pediatrics, Radboud University Medical Center Nijmegen, Geert Grooteplein Zuid 10, 6500 HB Nijmegen, The Netherlands

**Keywords:** Mitochondrial diseases, OXPHOS, Redox metabolism, Children, Sonlicromanol, GMFM, Clinical study protocol, KH176

## Abstract

**Background:**

**Methods:**

The KHENERGYC trial will be a phase II, randomised, double-blinded, placebo-controlled (DBPC), parallel-group study in the paediatric population (birth up to and including 17 years). The study will be recruiting 24 patients suffering from motor symptoms due to genetically confirmed PMD. The trial will be divided into two phases. The first phase of the study will be an adaptive pharmacokinetic (PK) study with four days of treatment, while the second phase will include randomisation of the participants and evaluating the efficacy and safety of sonlicromanol over 6 months.

**Discussion:**

Effective novel therapies for treating PMDs in children are an unmet need. This study will assess the pharmacokinetics, efficacy, and safety of sonlicromanol in children with genetically confirmed PMDs, suffering from motor symptoms.

**Trial registration:**

clinicaltrials.gov: NCT04846036, registered April 15, 2021. European Union Clinical Trial Register (EUDRACT number: 2020–003124-16), registered October 20, 2020. CCMO registration: NL75221.091.20, registered on October 7, 2020.

## Background

Mitochondria, being the energy generators of the cell, are involved in intermediary metabolism via several pathways, a major one being oxidative phosphorylation (OXPHOS) [1–4]. Energy generation (Adenosine triphosphate (ATP)) through OXPHOS comprises more than 80% of the cell requirement and denotes the most important function of this organelle [4–6].

Primary mitochondrial diseases (PMDs) are clinically heterogeneous metabolic disorders occurring due to disruption of the mitochondrial function [7]. This disruption may be attributed to genetic mutations in either nuclear deoxyribonucleic acid (nDNA) or mitochondrial DNA (mtDNA), causing a decrease in one or more OXPHOS enzyme complexes [1]. PMDs exist in several phenotypes along with a wide variety of clinical symptoms. These symptoms include cognitive decline, mental retardation, epilepsy, migraine, perceived fatigue, exercise intolerance, cardiomyopathy, conduction abnormality, diabetes mellitus, myopathy, and stunted growth [1]. Leigh Disease, MELAS (Mitochondrial Encephalomyopathy, Lactic acidosis, and Stroke-like episodes), MIDD (Maternally Inherited Diabetes and Deafness), and LHON (Leber’s Hereditary Optic Neuropathy) are some of the PMDs commonly observed [8]. Leigh syndrome is the most common PMD prevalent among children and may involve more than 75 gene mutations in the OXPHOS system [9]. The mitochondrial DNA 3243A > G mutation in the MT-TL1 gene is the most observed PMD defect in adults. However, the age of onset of the disease has been reported already in young children [1, 10, 11]. This syndrome is often persistently progressive, resulting in substantial morbidity and mortality. Further, this mutation is not limited to classical MELAS syndrome and may include other phenotypes like MIDD, chronic progressive external ophthalmoplegia (CPEO) and mixed phenotypes [1], all together described as MELAS spectrum disorders or alternatively m.3243 A > G MELAS with strokes and m.3243A > G non-MELAS (minus strokes) [12, 13].

Further, genetic defects of the OXPHOS system’s subunits often lead to increased generation of reactive oxygen species (ROS), causing abnormalities in the redox-controlled cell signalling in addition to irreversible damage to macromolecules via lipid peroxidation and protein carbonylation. It also shifts the cellular redox equilibrium to a more oxidising environment. Since oxidative distress and disparity in redox regulation are common pathological features in PMDs and play a significant role in the development of its clinical manifestations, they denote a crucial target for the advancement of therapy [14]. Regardless of advances in understanding these disorders, there are only a few treatment options available, and they are mostly supportive. Hence, there is an unmet need for novel treatment.

Small molecules targeting ROS may help to improve the regulation of cellular energy metabolism and, thus, be effective in PMDs. Such antioxidants, either in clinical use or clinical trials, include glutathione, N-acetylcysteine, lipoic acid, vitamin C, vitamin E, coenzyme Q10, Idebenone, MitoQ, etc. [15]. Certain redox-active molecules are also being studied in PMDs, such as EPI-743 (vatiquinone) [16], JP4–039 [17], SKQ1 [18] and KL1333 [19]. EPI-743 may halt disease progression and/or reversal in children with genetically confirmed Leigh syndrome in an open-label study [16]. JP4–039 has shown ROS scavenging effects in animal models and tumour cell lines [20, 21]. Further, SKQ1, a mitochondria-targeted antioxidant, has been studied in a mouse PMD model [18]. KL-1333, an NAD^+^ modulator has been shown to increase ATP levels and decrease ROS and lactate levels in human MELAS fibroblasts [19]. Future explorations of these small molecules may result in effective answers to the treatment of PMDs.

Sonlicromanol (KH176) is a small, orally available molecule being developed to treat PMDs. It has strong radical trapping activity in the cell, efficiently reduces increased levels of cellular ROS, and protects OXPHOS deficient cells from ROS-induced cell death. Furthermore, by targeting and activating the thioredoxin/peroxiredoxin enzyme machinery, it restores the redox balance. Sonlicromanol’s triple mode of action finally includes the specific inhibition of the microsomal PGES-1 enzyme explaining its anti-inflammatory properties [22]. It provides a novel approach for PMDs treatment due to its reductive and oxidative distress modulating, and anti-inflammatory properties [14, 22]. The EMA has granted sonlicromanol “Orphan Medicinal Drug Designation” to treat Leigh disease, MIDD, and MELAS syndrome. It has also received Orphan Drug Designation to manage all forms of inherited mitochondrial respiratory chain disorders and Rare Paediatric Disease (RPD) label to treat paediatric patients with MELAS syndrome by the US Food and Drug Administration (FDA).

A phase I placebo-controlled trial showed sonlicromanol to be well tolerated up to 800 mg (single dose or up to 400 mg in multiple doses). Its pharmacokinetics (PK) demonstrated a rapid absorption profile with a time to reach maximum concentration (T_max_) of about 2 h. The drug has a half-life of about 9 h, and its active metabolite has a half-life of about 15 h. Steady-state concentrations are achieved within three days of dosing [23]. Subsequently, a Phase IIa randomised, double-blind placebo-controlled trial (KHENERGY Study) was conducted with sonlicromanol in adults with confirmed mtDNA m.3243A > G mutation in transfer RNA^Leu(UUR)^. The results showed adequate safety and tolerability of twice-daily oral dosing of 100 mg of sonlicromanol. Importantly, indications of a possible treatment effect were observed on parameters relating to cognition and mood/depression. Present ongoing trials: Phase IIb (CTRI: NCT04165239) and 1-year open-label extension study (CTRI: NCT04604548) may confirm the efficacy of sonlicromanol in adult patients with PMDs [24].

As PMDs frequently occur in the paediatric population, there is a high need for effective treatments. For this reason, the paediatric study with sonlicromanol presented in this work was initiated relatively early in the development program for sonlicromanol. In line with the previous studies, the present study is designed to evaluate the PK, safety, and efficacy of sonlicromanol in children (aged from birth up to and including 17 years) who have genetically confirmed PMDs and suffering from motor symptoms.

## Methods

### Study design and overview

**Fig. 1:**
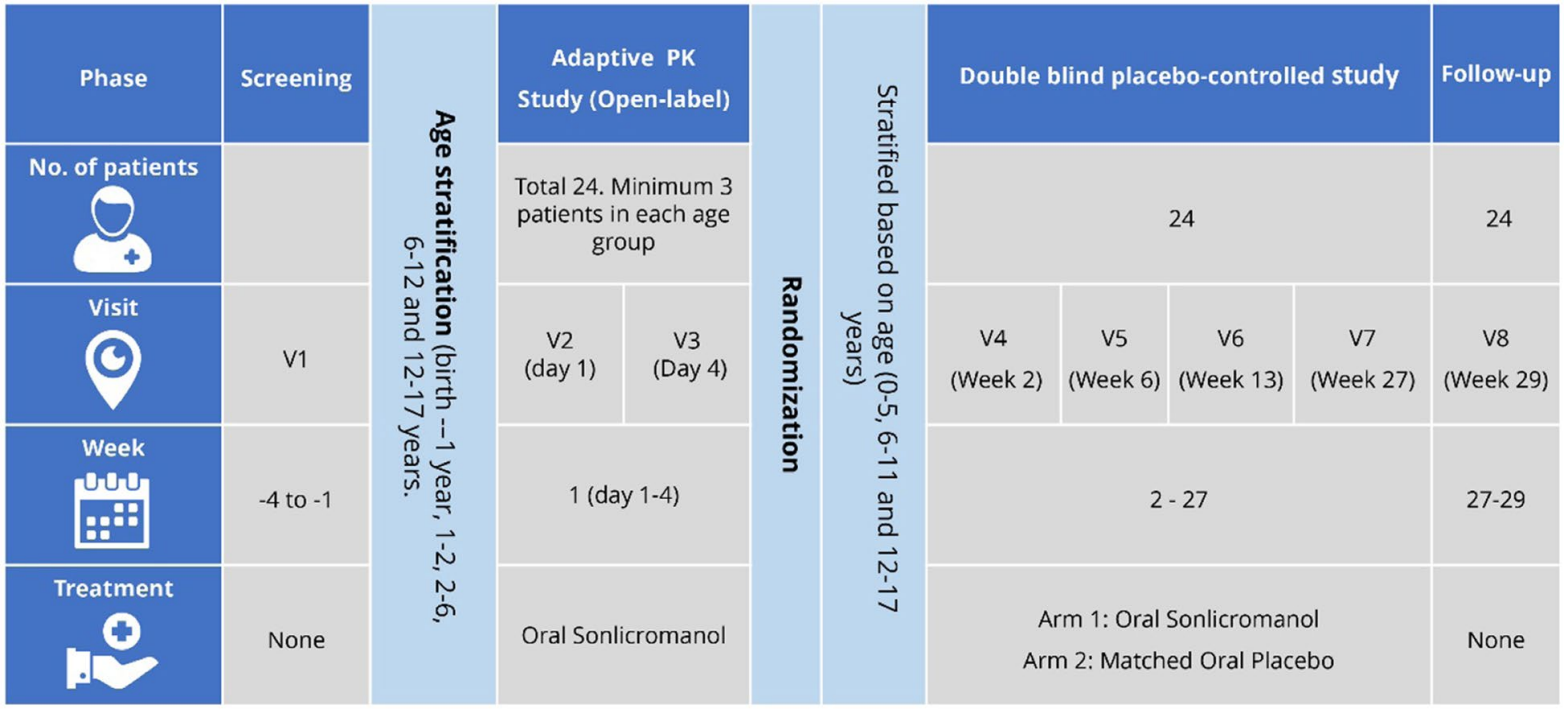
Study design

The KHENERGYC trial is a phase II, randomised, double-blinded, placebo-controlled (DBPC), parallel-group study to evaluate the safety, efficacy, and pharmacokinetics of sonlicromanol among the paediatric population with PMD and motor symptoms. The trial will be divided into two phases. The first phase of the study will be a 4-day adaptive pharmacokinetic (PK) study, while the second phase will include randomisation of the participants and evaluating the efficacy and safety of sonlicromanol over a 6-month period. The study design is depicted in Fig. 1. This study will be conducted at the Radboud University Medical Centre, Nijmegen, Netherlands. Approval of the trial was obtained from the local ethics committee (NL75221.091.20). The trial has been registered at the United States trial registry (clinicaltrials.gov: NCT04846036) as well as the European Union Clinical Trial Register (EudraCT number: 2020–003124-16).

During the first phase of the trial, four days of treatment will be provided to the children recruited in the following age groups: birth to 1 year, 1–2 years, 2–6 years, 6–12 years, and 12–17 years. A minimum of three participants will be enrolled in each age group. All participants will be provided with oral sonlicromanol for four days with the anticipated paediatric-equivalent dose (PED) (i.e., the dose which results in similar plasma sonlicromanol concentrations to adult patients with PMDs who receive a dose of 100 mg BID) from a Physiologically Based Pharmacokinetic analysis (PBPK model, data on file). The PK data obtained after completion of this phase will be analysed to confirm the PED to be used in the second phase of the study. Elder age groups will be studied before their younger counterparts.

The second phase of the trial will be the randomised DBPC study. It will involve age-based stratification of the participants and randomisation into treatment and placebo groups. The trial participants, caretakers, as well as the researchers will be blinded to the allocation of the study group. The treatment group will receive oral sonlicromanol (PED) twice daily, while the placebo group will be given a matching placebo treatment twice daily for 26 weeks (6 months). To avoid unblinding due to the bitterness of sonlicromanol, 50 mg or 100 mg mannitol containing 0.5 μg or 1 μg Bitrex^R^ (denatonium benzoate) will be added to the placebo formulation. Unblinding will be permissible only on the occurrence of serious or unexpected adverse events. A follow-up visit will be planned two weeks after the last dose of the treatment period. Each participant will receive a minimum of 178 days of treatment in the DBPC period.

### Study objectives

#### Primary objective

To assess the efficacy of sonlicromanol on the gross motor function over 6 months in children with genetically confirmed mitochondrial defects that affect the OXPHOS process.

#### Secondary objectives

To assess the effects of sonlicromanol over six months on growth (weight, height), fine manual motor skills, physical performance, spasticity, dystonia, ataxia, disability, signs, and symptoms of PMD, the burden on the caregiver, quality of life, clinician-scored and patient/caregiver scored global impression of change and patient/caregiver-identified 3 most bothersome symptoms of PMD.

#### Safety objectives

To assess the safety of sonlicromanol by assessing Treatment-emergent adverse events (TEAEs), vital signs (weight, temperature, systolic and diastolic blood pressure [SBP/DBP], heart rate [HR]), laboratory tests (chemistry, haematology), and electrocardiogram (ECG) will be monitored to evaluate the safety and tolerability over 6 months.

#### Additional objectives

To establish the PED of sonlicromanol and examine the multidose PK of sonlicromanol along with its active metabolite (KH176m) after 4 days and after 6 months; to analyse urine and blood samples for biomarkers (including the percentage of mtDNA heteroplasmy in urinary epithelial cells in patients with a mutation of mitochondrial transfer ribonucleic acid, fibroblast growth factor-21, growth/differentiation factor-15, and metabolomics endpoints); to collect information on Health Economics and Outcomes Research (HEOR) parameters, and to examine the palatability and acceptability of sonlicromanol.

### Outcome measures

#### Clinical efficacy outcome measures

All clinical efficacy assessments will be performed at all visits in the DBPC study. They will be conducted before the first dose, during V4, V5, V6, V7, and at follow-up (V8) of the trial. The testing procedure and timing of the assessments will be standardised as much as possible. The same rater/trial staff member will perform the assessments for clinician-reported assessments, wherever needed. All the measurements conducted for achieving the primary, secondary, and additional objectives are listed in Table 1.

**Table 1:** Outcome measures to be employed for evaluating the effect of sonlicromanol on the study objectives

Objective	Outcome measure	Comments	Reference
***Primary outcome measure***			
Gross motor symptoms of the genetic PMD in children	Gross Motor Function Measure −88 (GMFM-88)	• 88 items, 5 categories: lying and rolling; sitting; crawling and kneeling; standing; walking, running, and jumping. • 4-point Likert scale.	[25]
***Secondary outcome measures***			
Fine manual dexterity	9-Hole Peg Test (NHPT)	• Practice session at the screening visit.	[26]
The physical ability of the patient	10 Meter Walk Test (10MWT)	• 7-point scale ranging from total assistance to no assistance for walking/running.	[27, 28]
Muscle spasticity	Modified Tardieu Scale for Spasticity (MTS)	• Quality (on a 5-grade scale) and angle of muscle reaction.	[29]
Dystonia	Barry-Albright Dystonia Scale (BAD)	• 8 body parts will be evaluated: eyes, mouth, neck, trunk, and four limbs. • 5-point ordinal scale.	[30]
Ataxia	Scale for the Assessment and Rating of Ataxia (SARA)	• 8 items: gait, stance, sitting, speech, finger-chase test, nose-finger test, fast alternating movements, and heel-shin test.	[31]
Disability	Paediatric Evaluation of Disability Inventory (PEDI-CAT)	• Interview-based • 3 main domains: responsibility (51 items, 5-point scale), mobility (75 items), and social/cognitive ability (60 items, 4-point scale).	[32]
PMD signs and symptoms	International Paediatric Mitochondria Disease Scale (IPMDS)	• 3 domains of the disease: complaints and symptoms (23 items), physical examination (21 items), and functional tests (13 items, some are only for children ≥6 years).	[33]
Caregiver’s burden	ZARIT-12 Burden Scale	• Ranges from ‘not at all’ to ‘extremely’.	[34]
Quality of life	Neuro Quality of life Fatigue-Short Form (NeuroQL-SF) Paediatric version	• Physical, mental, and social effects. • 8-item self-assessment questionnaire recommended for children of 8–17 years.	[35, 36]
Clinician’s perception or improvement or worsening of the PMD	Clinician-scored Global Impression of Change (CGIC)	• 7-point Likert scale.	[36, 37]
Patient’s perception of change due to intervention	Patient/Caregiver scored Global Impression of Change (PGIC)	• 7-point Likert scale. • Will be completed by 12–18-year-old children. • For children younger than 12 years or cognitively disabled, parents/caregivers will be completing the test.	[36, 38]
Patient/Caregiver scored impression of change on patient-identified 3 most bothersome symptoms caused by PMD	Most Bothersome Symptom Assessment (MBSA)	• 7-point Likert scale. • Will be completed by 12–18-year-old children. • For children younger than 12 years or cognitively disabled, parents/caregivers will be completing the test.	[36]
Growth	Growth	• Overall body growth will be measured (height, weight, head circumference, and weight-for-height).	
***Additional outcome measures***			
Health Economics and Outcomes Research	EQ-5D-Y (Proxy 1)	• HRQoL via 5 dimensions: mobility, self-care, daily activities, pain/discomfort, and anxiety/depression. • Each dimension will have 5 levels: no, slight, moderate, severe, and extreme problems.	[39, 40]
	Health Utilities Index (HUI)	• Comprehensive health status and HRQoL.	[41, 42]
Acceptability of sonlicromanol	Entry in the diary by the parent/caregiver	• Evaluation will be based on whether the patient has swallowed the complete dose, spat out part of the dose, or refused to take the dose.	[43]
Palatability of sonlicromanol	Facial Hedonic Scale/Visual Analogue Scale-5 (FHS/VAS-5)		[43]

*PMD* Primary Mitochondrial Diseases, *GMFM-88* Gross Motor Function Measure −88, *NHPT* 9-Hole Peg Test, *10MWT* 10 Meter Walk Test, *MTS* Modified Tardieu Scale for Spasticity, *BAD* Barry-Albright Dystonia Scale, *SARA* Scale for the Assessment and Rating of Ataxia, *PEDI-CAT* Paediatric Evaluation of Disability Inventory, *IPMDS* International Paediatric Mitochondria Disease Scale, *NeuroQL-SF* Neuro Quality of life Fatigue-Short Form, *HRQoL* health-related quality of life, *CGIC* Clinician-scored Global Impression of Change, *PGIC* Patient/Caregiver scored Global Impression of Change, *MBSA* Most Bothersome Symptom Assessment, *HUI* Health Utilities Index, *FHS/VAS-5* Facial Hedonic Scale/Visual Analogue Scale-5, *EQ-5D-Y* EuroQol 5D youth version proxy version 1

#### Safety outcome measures

Vital signs, SBP/DBP, HR, ECG will be recorded before the first dose during all the visits (V1-V8) before and on PK-assessment days (just before taking a PK sample). Clinically significant abnormal findings in ECG will be recorded as adverse events (AE). A 2D echocardiography (left ventricular ejection fraction, left ventricular wall thickness, and left atrium dilatation) will be performed during the screening visit and at week 27.

Blood and other biological samples will be collected before the first dose at screening (V1) and V3-V8, in recommended volume based on the patient’s age and weight. Haematology and clinical chemistry assessments will be conducted to assess any TEAE. Depending on the event, follow-up may require additional tests or medical procedures as indicated and/or referral to the general physician or a medical specialist. Serious adverse events (SAEs) will be reported until the end of the study.

### Study participants

The study will be conducted in children (from birth up to and including 17 years) suffering from motor symptoms due to genetically confirmed PMD for which the gene defect is identified to decrease one or more OXPHOS system enzymes. Twenty-four (24) patients are planned to be randomised in the study.

#### Inclusion criteria

The study population will be children (from birth up to and including 17 years) suffering from genetic PMD for which the gene defect is identified to reduce one or more OXPHOS enzymes; with abnormal gross motor function and/or at least one of the clinically significant motor symptoms (hypotonia, dystonia, reduced muscle power, ataxia, chorea and/or spasticity) depending on the judgement by the investigator; having a score of ≤96% in Gross Motor Function Measure-88 (GMFM-88) and ≥ 10 in the International Paediatric Mitochondria Disease Scale (IPMDS), before allotment in the adaptive PK phase and randomisation into DBPC phase; with stable symptoms since the previous routine control visit based on the judgement by the investigator; those who will provide written informed (patient/parental/caregiver) consent, co-consent (consent provided by children aged between 12–16 years, in addition to consent from the parent/caregiver) or assent in addition to consent from the parent/caregiver (subjects aged <12 years) and who are able and willing to abide with the requirements of the study protocol. Further, only those women of childbearing age (WOCBP) who will be willing to use highly effective contraception methods for the duration of the study will be included. Male subjects with female partners of childbearing potential must be willing to use condoms.

#### Exclusion criteria

The exclusion criteria of the study are described below:

- Participants who have undergone gastrointestinal surgery with removal of stomach or duodenum/jejunum segments that interfere with absorption (feeding via gastrostomy tube will be permitted).
- Prior treatment with an investigational drug within 3 months or 5 times the half-lives of the investigational drug (whichever is longer) before the first dose of the study drug.
- Clinically significant cardiovascular disease or risk factors for arrhythmia based on judgement by the investigator like
  - Abnormal ECG (including QTcF exceeding the 95th percentile for the age- and sex-dependent QTc interval) and/or
  - Abnormality detected in 2D ECHO.
  - Systolic Blood Pressure (SBP) above the 95th percentile for the sex, age group, and height percentile at screening or baseline on a single measurement.
  - Any history of acute/chronic heart failure.
  - History of unexplained syncope.
  - Family history or medical history of congenital long and short QT syndrome or sudden death.
  - Hyper- or hypo-kalaemia; hyper- and hypomagnesaemia; hyper or hypocalcaemia.
- Clinically significant abnormal laboratory findings such as
  - Aspartate aminotransferase (ASAT), alanine aminotransferase (ALAT), or bilirubin >3 times the upper limit of normal (ULN) (If ASAT or ALAT >3 times but <3.5 times the ULN, re-assessment will be performed at the investigator’s decision).
  - Estimated glomerular filtration rate (eGFR) (based on the formula: 40.9*((1.8/Cystatine C) ^0.93^) below age-appropriate limits: < 2 months: < 25 ml/min/1.73 m^2^, 2 months to 1 year: < 35 ml/min/1.73 m^2^, > 1 year: < 60 ml/min/1.73 m^2^.
  - The investigator will judge other clinically significant parameters at screening or baseline.
- History of hypersensitivity to any of the components of the investigational drug.
- Medical history of drug abuse (use of narcotic agents such as cannabinoids, amphetamines, cocaine, opiates, or misuse of prescription drugs such as benzodiazepines, opiates).
- The use of any drug and/or supplements within 4 weeks or 5 times the half-life (whichever will be longer) before the first dose of the study drug, unless stable for at least one month before first dosing and remaining stable throughout the study. These includes
  - (Multi)vitamins, coenzyme Q10, vitamin E, riboflavin, and antioxidant supplements (including, but not limited to idebenone/EPI-743, mitoQ).
  - Any medication that influences the mitochondrial functioning negatively (including but not limited to valproic acid, glitazones, statins, anti-virals, amiodarone, and non-steroidal anti-inflammatory drugs),
  - Any strong cytochrome P450 (CYP)3A4 inhibitors (all conazoles-anti-fungal, HIV antivirals, grapefruit) or CYP3A4 inducers (including HIV antivirals, carbamazepine, phenobarbital, phenytoin, rifampicine, St. John’s wort, pioglitazone, troglitazone).
  - Any drug known to disturb cardiac repolarisation, unless the QTc interval at screening is normal during stable treatment for a period of two weeks, or 5 half-lives of the drug and its major metabolite(s), whichever is shorter (all antipsychotics, certain anti-depressants such as nortriptyline or amitriptyline, fluoxetine, anti-emetics like domperidone, granisetron, ondansetron).
  - Any drug metabolised by CYP3A4 with a narrow therapeutic index.

### Study procedures

#### Screening procedure

Informed consent by the parent/guardian, informed consent by the children older than 16 years, and child co-consent and assent (wherever applicable) will be obtained before the screening. The potential patients will be screened for the eligibility criteria (inclusion and exclusion criteria), including the medical history, physical examination (vital signs and cardiac health), clinical chemistry, haematology, and beta-human chorionic gonadotropin (for assessing pregnancy, WOCBP only). Further, 9 Hole Peg Test training will also be performed to reduce the testing burden at subsequent visits. Parents/caregivers will be provided diaries to keep daily records of study medication, seizures, or headache/migraine frequency. The details are mentioned in Table 2.

**Table 2:** Summary of study procedures

Study Period	Screening	Adaptive PK study		Double-blind Treatment Period				Follow up
Timing (weeks)	−4 to −1	Day 1	Day 4	2 (pre-dose) – 27				27–29
*Window (days)*				+/−7 (except +7 for V7)				+/− 2
Visit number	V1	V2	V3	V4^a^	V5	V6	V7^b^	V8
Day/Week	−4 to −1	Day 1	Day 4	Wk 2 / Day 1	Wk 6	Wk 13	Wk 27	Wk 29
Informed Consent	x							
Inclusion/Exclusion criteria	x	x		x				
Demographics	x			x^c^				
Medical history	x	x		x^c^				
Laboratory^d^	x		x	x^e^	x	x	x	x
Pregnancy test^f^	x	x	x	x	Monthly			
Physical examination	x			x	x	x	x	x
Bodyweight and height^g^	x			x	x^h^	x^h^	x	
Vital signs^i^	x	x	x^g^	x	x	x	x	x
ECG^k^	x	x^j^	x^j^	x	x	x	x	x
2D-Echocardiography^l^	x						x	
GMFM-88	x			x	x	x	x	x
IPMDS	x			x	x	x	x	x
Study medication		Daily						
Randomisation				x^m^				
PEDI-CAT				x^m^	x	x	x	x
BAD				x^m^	x	x	x	x
Tardieu Spasticity test				x^m^	x	x	x	x
SARA				x^m^	x	x	x	x
9 Hole Peg Test	x^n^			x^m^	x	x	x	x
10 MWT				x^m^	x	x	x	x
Zarit-12 Burden scale				x^m^	x	x	x	x
NeuroQL-SF				x^m^	x	x	x	x
Clinician-scored and Patient/Caregiver scored GIC, MBSA^o^				x	x	x	x	x
EQ-5D-Y				x^m^	x	x	x	x
Health Utilities Index				x^m^	x	x	x	x
Palatability		x						
Acceptability		Continuously						
PK sampling^p^		x	x				x	
Telephone compliance Checks^q^					Weekly			
AE recording		Continuously						
Concomitant medication		Continuously						
Diary^r^		Continuously						
Study medication dispensing		x		x	x	x		
Return study medication and Drug accountability			x		x	x	x	

^a^Visit not earlier than 10 days after the last dose in the adaptive PK study phase to avoid carry-over effects

^b^Assessments are also to be performed in case of premature discontinuation

^c^Demographics, Medical history on V4 only for patients not participating in the Adaptive PK study

^d^Including haematology and clinical chemistry parameters. Metabolomics and biomarkers in plasma and urine (overnight sampled portion or first-morning urine portion to be collected before early morning food/drinks intake) at V4 and V7. For patients with heteroplasmic mitochondrial DNA mutations: mtDNA heteroplasmy assessment in urine at V4 and V7

^e^Only for patients with heteroplasmic mitochondrial DNA mutations: mtDNA heteroplasmy assessment in urine at V4

^f^In females with childbearing potential only, as defined in section 3.2.9. Pregnancy blood test at screening, urine (dipstick) tests at monthly intervals, and at the Follow-up visit. Females of childbearing potential will be provided with urine (dipstick) pregnancy tests and will be instructed to perform the pregnancy tests at home, at monthly intervals throughout the double-blind study treatment period. The female subjects (or parent/caregiver) will be contacted by the study staff each month to report the results of the pregnancy tests

^g^For subjects <3 years: height, weight, skull circumference, and weight-for-height will be assessed. For subjects>3 years, weight, height, and BMI will be assessed

^h^Bodyweight only

^i^Including supine blood pressure and heart rate. Vital signs are to be recorded as close as possible to each PK assessment at V2, 3, and 7

^j^As close as possible before each sampling timepoint of the PK assessment

^k^ECGs will be recorded at Screening, Day 1 (V2) before first dosing on day 1; on Day 4 (V3) and Visit 7 just before the PK sample assessments; at day 1 (V4), at month 1 (V5), month 3 (V6), at month 6 (V7, just before the PK sample assessments) and Follow-up (V8)

^l^Not to be done if documented (favourable) result dated less than 6 months prior to screening is available

^m^Before trial, medication intake

^n^This is a training session to reduce the learning effect. By conducting the learning session at the screening visit, the testing burden at V4 is reduced

^o^This includes the Patient / Caregiver scored global impression of change for the patient/caregiver chosen 3 most bothersome symptoms. Patient scored global impression of change to be assessed by parent/caregiver for all children under 12 years of age and children aged 12–18 considered unable to provide a reliable assessment. A baseline situation is recorded to document the most important signs and symptoms to base the impression of change

^p^On days 1 and 4 and Visit 7, the PK sampling schedule depends on age

^q^Telephone contacts will be conducted starting from Day 1 of the double-blind treatment period to verify the subject’s compliance with medication intake and the correct completion of the daily diaries. In addition, female subjects of childbearing potential and/or parent/caregiver will be contacted each month and asked to report the results of the urine (dipstick) pregnancy tests to confirm the absence of pregnancy

^r^Parent/caregiver of the subject will keep a diary during the study, for daily recording of intake of study medication, including seizures, migraine frequency

#### Adaptive PK study procedure

After screening, subject eligibility will be reconfirmed on the Day 1 of V2, before the first dose of the study medication. The blood sampling for PK studies will be done at V2 (Day 1) and V3 (Day 4). The amount of blood withdrawn will depend on the age and weight of the patient and mostly follow the 5-sample schedule for PK studies. Further, the blood samples will also be used for safety laboratory assessments (haematology and clinical chemistry tests) (Table 2).

The patients will be enrolled in specific age groups (birth to 1 year, 1 to 2 years, 2 to 6 years, 6 to 12 years, and 12 up to and including 17 years) (Fig. 1). The PK and safety data of the patients (at least 3 patients) in each age group will be analysed to establish the paediatric-equivalent dose (PED) that will be used in the DBPC phase of the study. After completion of the PK assessments and analysis per age group (minimum 3 patients), a Data and Safety Monitoring Board (DSMB) will review the safety and PK data. It will issue an official recommendation on the safety of the study subjects and the proposed dose in the specific age group. This may lead to a change in sonlicromanol dose for one or more age groups. This DSMB recommendation letter will be submitted to the Medical Research Ethics Committee (MREC) for approval. After receiving MREC’s approval on the DSMB recommendation, the patients in the specific age group will be enrolled in the DBPC phase of the study. The adaptive PK study with the anticipated PED will be initially conducted in the elder children: older age groups will be studied before younger age groups.

#### Double-blind, placebo-controlled phase

All the patients completing the first phase of the study (the Adaptive PK study) will be offered participation in the DBPC phase. When a minimum of three subjects in each age cohort has completed the Adaptive PK phase, new participants from that age group may be enrolled, directly in the DBPC phase without having to participate in the Adaptive PK study.

In this second phase, subjects will be randomised (by age group) in a 1:1 ratio (using block size of 4) over two arms using *Lifesphere* EDC and *Lifesphere* Central Coding (vendor: *ArisGlobal*) [44] The allocation of randomization sequence is fully integrated into the *LifeSphere* EDC system and is blinded to the patient, caretaker and the researcher. The randomization number will be automatically generated by *Lifeshpere* EDC [44] and will be used for assigning the subjects to the treatment groups. Arm 1 will receive oral sonlicromanol (PED) twice daily for 26 weeks (6 months), while Arm 2 will receive a matching placebo twice daily for 26 weeks (6 months). The patients and/or parents/caregivers will be required to maintain the patients’ daily diaries. The patients’ eligibility will be reconfirmed on Day 1 of the treatment period (V4) before randomisation. Further, the patients will be assessed for all the clinical and safety outcome measures at each visit during the treatment phase. Table 2 describes the detailed study procedures. A final follow-up visit (V8) will be scheduled two weeks after the intake of the last dose of the treatment period. Study medication will be collected at each visit, after which, new study medication kits will be provided. All the remaining study medications will be collected during the final visit.

#### Withdrawal procedure

Premature discontinuation from the study will occur whenever the participant does not finish the scheduled 26 weeks treatment period and the follow-up assessment. The participants will be allowed to prematurely terminate the study at any time or be excluded from the study at the investigator’s decision if continuous administration of the study drug is thought not to be in the participant’s best interests. Also, the participant will be discontinued by the investigator or the sponsor (designee) if enrolment into the study is found inappropriate, the study plan is violated, and/or for any other safety reasons. Mandatory withdrawal criteria will be applicable when QTc exceeds the 95th percentile for the age- and sex-dependent QTc interval or if female participants become pregnant. Withdrawal of the assent/(co-)consent by subject/parents/caregiver or loss to follow-up will also lead to premature termination. Failure or refusal of blood sampling for the PK assessment at Day 4 (V3) of the PK study will also be treated as a withdrawal of consent and result in termination of the participant.

### Study medication

The medicinal product under investigation is sonlicromanol, administered as an oral liquid. It will be manufactured and provided under the responsibility of the sponsor. Sonlicromanol, as well as the placebo, will be provided as a powder for oral solution. To maintain the blinding of the study, the placebo formulation will be matched to the active study medication in size, colour, shape, and taste. The investigational medicinal product and its matching placebo will be packaged in the same way, in glass bottles according to a randomisation list.

#### Route of administration and Dosage

Study medications (sonlicromanol and placebo) will be made available as a powder that must be dissolved in water. An oral syringe will be used to administer the solution in the correct volume. Patients with a nasogastric/ percutaneous endoscopic gastrostomy tube will be dosed through the stoma. The first daily dose should be taken in the morning, followed by the second after 12 h. A minimum 6-h allowable intake window will be provided, i.e., two doses must not be taken within 6 h of one another and not more than 18 h apart.

Sonlicromanol will be given in the anticipated PED for four days (Adaptive PK study), followed by sonlicromanol or placebo in the confirmed PED twice daily for 26 weeks (DBPC phase). According to Physiologically Based Pharmacokinetics (PBPK) model and the plasma concentration results from the PK phase, the anticipated PED will be administered in fixed doses, comparable in exposure to adults with PMDs treated at 100 mg, twice daily (Table 3).

**Table 3:** Anticipated Paediatric-equivalent dose comparable to adults with mitochondrial disease treated at 100 mg twice daily

Population	Dose (mg) per administration (BID)
Neonates (0–28 days)	2
Infants (1–2.5 months)	4
Infants (2.5–12 months)	12
Toddlers (1–2 years)	23
Young Children (2–6 years)	33
Middle-Aged Children (6–12 Years)	55
Adolescents (12–17 years)	80

### Power and sample size calculation

There is limited information on GMFM-88 scores in the target population [45–48]. Based on the available information, the sample calculations were conducted assuming the standard deviation (SD) range between 2 and 4.5% and an effect size of 4% or 5%. A sample size of 12 (in each group) was calculated to achieve a power of 80%. This could detect a mean change from a baseline difference of 4% between the groups for the total GMFM-88 score.

### Statistical analysis

All statistical analyses will be performed using the SAS® System (SAS Institute Inc., Cary, NC, USA). Continuous data will be presented using descriptive statistics where the parameters will be reported such as n (number of observations), mean, SD, minimum (Min), median and maximum (Max). Geometric means will be used for PK variables (AUC and C_max_). Absolute and relative frequencies will be utilised to analyse categorical variables. Unless otherwise specified, all tests will be two-sided at the 5% level, and all the confidence intervals will be two-sided at the 95% level. Given the number of assessments conducted in this trial, the SAP will include appropriate corrections for multiple testing using a hierarchical procedure and/or alpha correction.

Four different analyses sets will be applied for performing the statistical analysis, namely: all-randomised, all-treated, safety, and per-protocol (PP) sets. The populations used in the efficacy analyses of the DBPC phase will be the all-randomised and PP populations. The all-randomised population will consist of all patients randomised for the double-blinded treatment, regardless of whether they received the study medication or not, consistent with the Intention-to-Treat (ITT) principle. The primary efficacy endpoint (change in GMFM-88 score) at weeks 6, 13, 27, and 29 will be performed using the all-randomized and PP population. The PP population will consist of all patients in the all-treated set who will not violate the protocol in a way that could affect the primary outcome measure. The population for assessing the safety will consist of all patients in the safety set. This population will include all randomised patients receiving the study medication and for whom at least one safety assessment (after first drug intake) is available (patients are assigned the treatment actually received). Descriptive statistics will be used to analyse the safety data. The demographics, subject disease characteristics, and baseline data (including medical history) will be presented descriptively for the PK study (V1 or V2) and DBPC study (V4).

### Data collection, management and monitoring

All data are either transferred from third parties e.g., labs, ECG analysis provider, etc., or entered directly into the e-CRF by site personnel. Reference ranges are imported in the *Lifesphere* EDC [44]. Further, all the data from the database, external data, coded data, and any other derived data files used for the analysis of the clinical project will be stored on secure electronic storage media. The data management and monitoring will be performed using *Lifesphere* EDC and *Lifeshpere* Central Coding system [44]. Verbatim terms will be coded using the *LifeSphere* Central Coding system. However, manual coding will be done for terms that are not automatically coded by the *LifeSphere* Central Coding system and will be reviewed by a medical reviewer. Adverse events, medical history and concomitant treatment verbatim terms will be coded according to MedDRA® and WHO Drug Global thesauri. Further details of data management procedures are described in the Data Management Plan.

Moreover, the safety management plan will be in place for collecting, assessing, reporting, and managing solicited and spontaneously reported adverse events and other unintended effects of trial interventions or trial conduct. The periodic safety monitoring of the participants will be performed by DSMB committee consisting of 3 members, independent from the sponsor, without any competing interests. The DSMB will be providing its recommendations to the sponsor.

### Dissemination plans

The trial results will be communicated to participants, healthcare professionals, the public, and other relevant groups via a publication, or at the conferences/meetings.

## Discussion

Mitochondrial diseases are a group of disorders with no proven effective treatment to date, except for idebenone for LHON authorized under exceptional circumstances in the European Union (EU) [8, 49, 50]. Novel experimental strategies that fall into two broad categories, pharmacological and genetic approaches, are being investigated. Several recent reviews have focused on these emerging therapies [7, 51, 52]. Based on the current understanding of the effects of OXPHOS system flaws at the cellular level, several novel small molecules potentially capable of mitigating the consequences of hampered oxidative phosphorylation are in the preclinical developmental stage, and few of them have reached the clinical development stage [8, 15, 51, 53].

The prevalence of childhood-onset (age less than 17 years) PMDs ranges between 5–15 cases per 100,000 individuals [1]. Mitochondrial disease presenting in childhood is characterized by clinical, biochemical, and genetic complexity. Some children are affected by distinct syndromes, but the majority have nonclassical multisystemic disease presentations involving virtually any organ in the body. Each child has a unique constellation of clinical features and disease trajectory, leading to enormous challenges in the diagnosis and management of these heterogeneous disorders [8].

The study drug sonlicromanol has shown beneficial properties on motor coordination and motor learning in preclinical models of Leigh disease [54] and potential efficacy signals on brain involvement in adult patients with PMDs [24]. Since PMDs hinder the development of children, effective therapies for treating specific PMDs in children are urgently needed. The present study will help to characterise the pharmacokinetics, efficacy, and safety of sonlicromanol in children (up to 17 years old) with genetically confirmed PMDs and who suffer from motor symptoms.

The study has been initiated in February 2021 and is currently actively recruiting patients.

## Data Availability

Data sharing not applicable to this article as no datasets were generated or analysed during the current study. However, the datasets generated from the current study will be made available from the corresponding author upon reasonable request and in agreement with the research collaboration and data transfer guidelines of the UMC Nijmegen.

## Abbreviations

ALAT: Alanine aminotransferase
ASAT: Aspartate aminotransferase
ATP: Adenosine triphosphate
BAD: Barry-Albright Dystonia scale
DBP: Diastolic Blood pressure
DBPC: Double-blind, placebo-controlled
ECG: Electrocardiogram
e-CRF: Electronic Case Report Form
EMA: European Medicines Agency
EU: European Union
FDA: Food and Drug Administration
HEOR: Health Economics and Outcomes Research
HR: Heart rate
IPMDS: International Paediatric Mitochondrial Disease Scale
ITT: Intention-to-Treat
LHON: Leber’s Hereditary Optic Neuropathy
MELAS: Mitochondrial Encephalomyopathy, Lactic acidosis, and Stroke-like episodes
MIDD: Maternally Inherited Diabetes and Deafness
MREC: Medical Research Ethics Committee
mtDNA: Mitochondrial DNA
nDNA: Nuclear DNA
NeuroQL-SF: Neurology Quality of life short Form
OXPHOS: Oxidative phosphorylation
PED: Paediatric-equivalent dose
PEDI-CAT: Paediatric Evaluation of Disability Inventory
PMDs: Primary mitochondrial diseases
ROS: Reactive oxygen species
RPD: Rare Paediatric Disease
SAP: Statistical Analysis Plan
SARA: Scale for the Assessment and Rating of Ataxia
SBP: Systolic Blood Pressure
TEAEs: Treatment-emergent adverse events
ULN: Upper limit of normal

## References

[1] Gorman GS, Chinnery PF, DiMauro S, Hirano M, Koga Y, McFarland R (2016). Mitochondrial diseases. Nat Rev Dis Primers.

[2] Koopman WJ, Willems PH, Smeitink JA (2012). Monogenic mitochondrial disorders. N Engl J Med.

[3] Nunnari J, Suomalainen A (2012). Mitochondria: in sickness and in health. Cell..

[4] Papa S, Martino PL, Capitanio G, Gaballo A, De Rasmo D, Signorile A (2012). The oxidative phosphorylation system in mammalian mitochondria. Adv Exp Med Biol.

[5] Duchen MR (2004). Mitochondria in health and disease: perspectives on a new mitochondrial biology. Mol Asp Med.

[6] Schlieben LD, Prokisch H (2020). The dimensions of primary mitochondrial disorders. Front Cell Dev Biol.

[7] Tinker RJ, Lim AZ, Stefanetti RJ, McFarland R (2021). Current and emerging clinical treatment in mitochondrial disease. Mol Diagn Ther.

[8] Rahman S (2020). Mitochondrial disease in children. J Intern Med.

[9] Lake NJ, Compton AG, Rahman S, Thorburn DR (2016). Leigh syndrome: One disorder, more than 75 monogenic causes. Ann Neurol.

[10] Hirano M, Pavlakis SG (1994). Mitochondrial myopathy, encephalopathy, lactic acidosis, and strokelike episodes (MELAS): current concepts. J Child Neurol.

[11] Okhuijsen-Kroes EJ, Trijbels JM, Sengers RC, Mariman E, van den Heuvel LP, Wendel U (2001). Infantile presentation of the mtDNA A3243G tRNA(Leu (UUR)) mutation. Neuropediatrics..

[12] de Laat P, Rodenburg RR, Roeleveld N, Koene S, Smeitink JA, Janssen MC (2021). Six-year prospective follow-up study in 151 carriers of the mitochondrial DNA 3243 A>G variant. J Med Genet.

[13] Sharma R, Reinstadler B, Engelstad K, Skinner OS, Stackowitz E, Haller RG, et al. Circulating markers of NADH-reductive stress correlate with mitochondrial disease severity. J Clin Investig. 2021;131(2). 10.1172/jci136055. 10.1172/JCI136055 PMC7810486 33463549

[14] Beyrath J, Pellegrini M, Renkema H, Houben L, Pecheritsyna S, van Zandvoort P (2018). KH176 safeguards mitochondrial diseased cells from redox stress-induced cell death by interacting with the thioredoxin system/peroxiredoxin enzyme machinery. Sci Rep.

[15] Bottani E, Lamperti C, Prigione A, Tiranti V, Persico N, Brunetti D (2020). Therapeutic approaches to treat mitochondrial diseases: "one-size-fits-all" and "precision medicine" strategies. Pharmaceutics..

[16] Martinelli D, Catteruccia M, Piemonte F, Pastore A, Tozzi G, Dionisi-Vici C (2012). EPI-743 reverses the progression of the pediatric mitochondrial disease--genetically defined Leigh Syndrome. Mol Genet Metab.

[17] Frantz MC, Skoda EM, Sacher JR, Epperly MW, Goff JP, Greenberger JS (2013). Synthesis of analogs of the radiation mitigator JP4-039 and visualization of BODIPY derivatives in mitochondria. Org Biomol Chem.

[18] Shabalina IG, Vyssokikh MY, Gibanova N, Csikasz RI, Edgar D, Hallden-Waldemarson A (2017). Improved health-span and lifespan in mtDNA mutator mice treated with the mitochondrially targeted antioxidant SkQ1. Aging..

[19] Seo KS, Kim JH, Min KN, Moon JA, Roh TC, Lee MJ (2018). KL1333, a novel NAD(+) modulator, improves energy metabolism and mitochondrial dysfunction in MELAS fibroblasts. Front Neurol.

[20] Leipnitz G, Mohsen AW, Karunanidhi A, Seminotti B, Roginskaya VY, Markantone DM (2018). Evaluation of mitochondrial bioenergetics, dynamics, endoplasmic reticulum-mitochondria crosstalk, and reactive oxygen species in fibroblasts from patients with complex I deficiency. Sci Rep.

[21] Seminotti B, Leipnitz G, Karunanidhi A, Kochersperger C, Roginskaya VY, Basu S (2019). Mitochondrial energetics is impaired in very long-chain acyl-CoA dehydrogenase deficiency and can be rescued by treatment with mitochondria-targeted electron scavengers. Hum Mol Genet.

[22] Jiang X, Renkema H, Pennings B, Pecheritsyna S, Schoeman JC, Hankemeier T (2021). Mechanism of action and potential applications of selective inhibition of microsomal prostaglandin E synthase-1-mediated PGE(2) biosynthesis by sonlicromanol's metabolite KH176m. Sci Rep.

[23] Koene S, Spaans E, Van Bortel L, Van Lancker G, Delafontaine B, Badilini F (2017). KH176 under development for rare mitochondrial disease: a first in man randomized controlled clinical trial in healthy male volunteers. Orphanet J Rare Dis.

[24] Janssen MCH, Koene S, de Laat P, Hemelaar P, Pickkers P, Spaans E (2019). The KHENERGY study: safety and efficacy of KH176 in mitochondrial m.3243A>G spectrum disorders. Clin Pharmacol Ther.

[25] Russell D, Rosenbaum P, Avery L, Lane M (2002). Gross motor function measure (GMFM-66 and GMFM-88) user's manual: clinics in developmental medicine.

[26] Smith YA, Hong E, Presson C (2000). Normative and validation studies of the Nine-hole Peg Test with children. Percept Mot Skills.

[27] Nagy S, Schmidt S, Hafner P, Klein A, Rubino-Nacht D, Gocheva V, et al. Measurements of motor function and other clinical outcome parameters in ambulant children with duchenne muscular dystrophy. J Vis Exp. 2019;143. 10.3791/58784. 10.3791/58784 30688316

[28] Tyson S, Connell L (2009). The psychometric properties and clinical utility of measures of walking and mobility in neurological conditions: a systematic review. Clin Rehabil.

[29] Boyd RN, Graham HK (1999). Objective measurement of clinical findings in the use of botulinum toxin type A for the management of children with cerebral palsy. Eur J Neurol.

[30] Barry MJ, VanSwearingen JM, Albright AL (1999). Reliability and responsiveness of the Barry-Albright Dystonia Scale. Dev Med Child Neurol.

[31] Schmitz-Hübsch T, du Montcel ST, Baliko L, Berciano J, Boesch S, Depondt C (2006). Scale for the assessment and rating of ataxia. Neurology..

[32] Dumas HM, Fragala-Pinkham MA, Haley SM, Ni P, Coster W, Kramer JM (2012). Computer adaptive test performance in children with and without disabilities: prospective field study of the PEDI-CAT. Disabil Rehabil.

[33] Koene S, Hendriks JCM, Dirks I, de Boer L, de Vries MC, Janssen MCH (2016). International Paediatric Mitochondrial Disease Scale. J Inherit Metab Dis.

[34] Bédard M, Molloy DW, Squire L, Dubois S, Lever JA, O'Donnell M (2001). The Zarit Burden Interview: a new short version and screening version. Gerontologist..

[35] Cella D, Lai JS, Nowinski CJ, Victorson D, Peterman A, Miller D (2012). Neuro-QOL: brief measures of health-related quality of life for clinical research in neurology. Neurology..

[36] Karaa A, Haas R, Goldstein A, Vockley J, Cohen BH (2020). A randomized crossover trial of elamipretide in adults with primary mitochondrial myopathy. J Cachexia Sarcopenia Muscle.

[37] Busner J, Targum SD (2007). The clinical global impressions scale: applying a research tool in clinical practice. Psychiatry (Edgmont).

[38] Hurst H, Bolton J (2004). Assessing the clinical significance of change scores recorded on subjective outcome measures. J Manip Physiol Ther.

[39] EuroQol. (1990). EuroQol--a new facility for the measurement of health-related quality of life. Health Policy.

[40] Herdman M, Gudex C, Lloyd A, Janssen M, Kind P, Parkin D (2011). Development and preliminary testing of the new five-level version of EQ-5D (EQ-5D-5L). Qual Life Res.

[41] Eiser C, Morse R (2001). The measurement of quality of life in children: past and future perspectives. J Dev Behav Pediatr.

[42] Horsman J, Furlong W, Feeny D, Torrance G (2003). The Health Utilities Index (HUI): concepts, measurement properties and applications. Health Qual Life Outcomes.

[43] Thompson C, Lombardi D, Sjostedt P, Squires L (2015). Best practice recommendations regarding the assessment of palatability and swallowability in the development of oral dosage forms for paediatric patients. Ther Innov Regul Sci.

[44] Lifesphere EDC system and Central Coding (2022). Vendor: Arisglobal.

[45] Fujii T, Nozaki F, Saito K, Hayashi A, Nishigaki Y, Murayama K (2014). Efficacy of pyruvate therapy in patients with mitochondrial disease: a semi-quantitative clinical evaluation study. Mol Genet Metab.

[46] Koene S, van Bon L, Bertini E, Jimenez-Moreno C, van der Giessen L, de Groot I (2018). Outcome measures for children with mitochondrial disease: consensus recommendations for future studies from a Delphi-based international workshop. J Inherit Metab Dis.

[47] Mancuso M, McFarland R, Klopstock T, Hirano M (2017). International Workshop: Outcome measures and clinical trial readiness in primary mitochondrial myopathies in children and adults. Consensus recommendations. 16–18 November 2016, Rome, Italy. Neuromuscul Disord.

[48] Sage-Schwaede A, Engelstad K, Salazar R, Curcio A, Khandji A, Garvin JH (2019). Exploring mTOR inhibition as treatment for mitochondrial disease. Ann Clin Transl Neurol.

[49] Liufu T, Wang Z. Treatment for mitochondrial diseases. Rev Neurosci. 2020; [published online ahead of print. 10.1515/revneuro-2020-0034. 10.1515/revneuro-2020-0034 32903211

[50] Lyseng-Williamson KA (2016). Idebenone: a review in Leber's hereditary optic neuropathy. Drugs.

[51] Viscomi C, Zeviani M (2020). Strategies for fighting mitochondrial diseases. J Intern Med.

[52] Wang Y, Hu LF, Zhou TJ, Qi LY, Xing L, Lee J (2021). Gene therapy strategies for rare monogenic disorders with nuclear or mitochondrial gene mutations. Biomaterials.

[53] Russell OM, Gorman GS, Lightowlers RN, Turnbull DM (2020). Mitochondrial diseases: hope for the future. Cell.

[54] de Haas R, Das D, Garanto A, Renkema HG, Greupink R, van den Broek P (2017). Therapeutic effects of the mitochondrial ROS-redox modulator KH176 in a mammalian model of Leigh Disease. Sci Rep.

